# Dr. Harris’s Circular Letter

**Published:** 1846-10

**Authors:** Chapin A. Harris

**Affiliations:** Baltimore


### DR. HARRIS'S CIRCULAR LETTER.

We take great pleasure in making room for the following circular addressed to his brethren by Dr. Harris, whose zeal in advancing his profession is worthy of all praise:—

Baltimore, August 22, 1846.

Dear Sir—The undersigned is engaged in preparing for publicaton a *Dictionary of Dental Science*, a work which is evidently much needed, and which he hopes to present in such a form as will commend it to the general use of the profession.

In the hope of eliciting information of an important character which your observation and experience may enable you to give, and with a desire to furnish you with an opportunity to make known any improvement in the dental art or discoveries in dental science, made either by yourself, or by others known to you, which have not yet been promulgated, the undersigned respectfully solicits an early answer to the following queries:

First. Have you knowledge of any deceased dentist or dentists, whose contributions to dental literature, superior skill, or remarkable character, entitle him or them to biographical notice in such a work as the one proposed? If so, the undersigned would be glad to have names and such items of history as your judgment may select. If your information concerning them will enable you to do so, state when and where they were born; the character of their early pursuits, the extent of their education, with whom they studied and served their professional apprenticeship; when they commenced practice; their skill in the several branches of the dental art, the improvements they made either in theory or practice, or in dental instruments; their contributions to the literature of dental science, the place or places where they practised; their standing in society, and when and where they died, with the diseases which caused their deaths.

Second. Have you invented any dental instrument or appliance of any kind, which upon full trial you consider valuable to the profession? If so, please describe it.

Third. Have you improved any instrument previously known? If so, please transmit a description of it.

Fourth. Have you performed any remarkable or extraordinary operation upon the mouth? If so, describe it, pointing out any particulars which entitle you to the award of originality in conception, or superior dexterity in operating. Do not confine your answer to operations on the teeth, but include the whole buccal cavity.

Fifth. Do you know of any such operation performed by any other than yourself, not yet reported?

Sixth. Have you met with any remarkable cases of disease or deformity of the organs in question? If so, describe them, with the mode of treatment adopted, and any other information with regard to them.

Seventh. Have you remarked serious results from the use of unscientific preparations, awkward operations? &c., &c.

Eighth. Have you made observations which you think valuable upon the causes of dental disease, and their prevention? If so, please transmit them in such form as you may think proper.

It would be very desirable, if you could do so conveniently, to accompany any description, which you may have the kindness to furnish, of any newly invented instrument or appliance, or of any improvement on any previously in use, with an accurate drawing.

By answering the above queries or any of them, you will confer a favor on the undersigned, and may render valuable service to science. Very respectfully, &c., &c.,

CHAPIN A. HARRIS.

